# Wellbeing Learnings from Pandemic Pedagogies in Aotearoa New Zealand

**DOI:** 10.1007/s40841-023-00278-3

**Published:** 2023-02-27

**Authors:** Jenny Ritchie

**Affiliations:** Te Herenga Waka Victoria University of Wellington, Wellington, New Zealand

**Keywords:** Te ao Māori, Aotearoa, New Zealand, COVID-19, Wellbeing, Education, Teaching

## Abstract

This paper discusses data from a survey of New Zealand teachers conducted in 2020 during the first months of the COVID-19 pandemic. It considers this data in the light of a series of contexts: Te Tiriti o Waitangi; social inequalities particularly in relation to the impacts of colonisation and neoliberal social and economic policies on Māori; the New Zealand government’s commitment to wellbeing; Te Ara Waiora, a Māori model of wellbeing utilised by the New Zealand Treasury; and the status of the teaching profession in Aotearoa New Zealand. Using data from the teachers’ responses to the survey, it outlines ways in which wellbeing was prioritised by teachers during these early months of the pandemic, when teachers were suddenly required to pivot to online teaching. It argues that the wellbeing values as espoused in te ao Māori, a Māori worldview, and those articulated by teachers provide inspiration for a pathway beyond the privations of the pandemic.

## Introduction

In early 2020, as the reach and severity of the global COVID-19 pandemic began to impact our lives and worlds as educators, a group of nine education researchers from four different countries (Australia, Singapore, USA, and Aotearoa New Zealand) came together in response to a call from Louise Phillips. Thus the Teaching and Learning in COVID Times study was initiated, and a Qualtrics survey disseminated in May, 2020 (L. Phillips et al., 2021a, 2021b). This paper considers the data from the 51 New Zealand teachers who participated in this survey. It explores these data in the contexts of the New Zealand government’s stated ‘wellbeing’ focus, underpinned by its obligations to Māori required by the 1840 Tiriti o Waitangi (Orange, 2021). This paper accepts the claim by notable Māori scholars that what is good for Māori is good for everyone in our education institutions and beyond (Durie, 2005; Penetito, 2011; Skerrett, 2010). It also recognises the significant role that teachers play in the lives and wellbeing of children and families, and problematises the undervaluing of the teaching profession. It considers learnings from this study in our particular Aotearoa (New Zealand) context that may enable us to reimagine education differently in the future.

One of the key insights that emerged almost immediately from government, community and educators’ responses in Aotearoa to the COVID-19 pandemic is that we have collectively and individually demonstrated the capacity to immediately, quickly and drastically change our everyday lives. As Bruno Latour explains: “The first lesson the coronavirus has taught us is also the most astounding: we have actually proven that it is possible, in a few weeks, to put an economic system on hold everywhere in the world and at the same time, a system that we were told it was impossible to slow down or redirect” (Latour, 2020, p. 1). Latour invites us to reconsider the globalised economic structures that are accelerating the destruction of our biosphere, by critiquing “each segment of this so-called irreversible system, putting a question mark over each of its supposed indispensable connections, and then testing in more and more detail what is desirable and what has ceased to be so” (p. 2).

Similarly, Arundhati Roy has posited the pandemic as a portal to a different future:

Whatever it is, Covid-19 has made the mighty kneel and brought the world to a halt like nothing else could. Our minds are still racing back and forth, longing for a return to ‘normality’, trying to stitch our future to our past and refusing to acknowledge the rupture. But the rupture exists. And in the midst of this terrible despair, it offers us a chance to rethink the doomsday machine we have built for ourselves. Nothing could be worse than a return to normality. Historically, pandemics have forced humans to break with the past and imagine their world anew. This one is no different. It is a portal, a gateway between one world and the next.

We can choose to walk through it, dragging the carcasses of our prejudice and hatred, our avarice, our data banks and dead ideas, our dead rivers and smoky skies behind us. Or we can walk through lightly, with little baggage, ready to imagine another world. And ready to fight for it. (Roy, 2020, p. 214)

This sudden rupture of the previously unquestioned, taken-for-granted ‘normality’ has liberatory potential in enabling us to think and act otherwise. As educators, our influence on children and families is profound. The values and priorities that we model and inculcate will have a long-standing influence. This paper draws on the voices of teachers to identify how they prioritised wellbeing, compassion and kindness during the first months of the 2020 covid lockdown restrictions. It signals how such values, compatible with a Māori worldview of wellbeing now recognised by the New Zealand government, can inform future pathways for education and society in general.

## COVID-19 and Wellbeing

Pandemics are recognised as exacerbating and intensifying “existing conditions of colonial inequality and injustice” (Barber & Naepi, 2020, p. 694) and this was certainly an outcome in Aotearoa (Estellés et al., 2022). The onset in early 2020 of the COVID-19 pandemic brought a huge and ongoing test for the New Zealand government with regard to the wellbeing of its citizenry, and in particular, those who were most vulnerable, such as those with insecure housing, employment and health situations, including disproportionate numbers of Māori and Pacific peoples (Houkamau et al., 2021). The government has been criticised for adopting a generic approach that fails to address disparities in access and provision of supports, along with a lack of support for Māori led health initiatives, a situation that has persisted since colonisation began, and which operates in breach of Te Tiriti o Waitangi (Pihama & Lipsham, 2020). Iwi Māori and Pacific peoples have responded by working collaboratively to protect and support whanau, particularly given their historic memories of the inequitable and severe impacts of the global influenza pandemic over a century ago (Cook et al., 2020). This work has been underpinned by core Māori values such as manaakitanga, and tikanga such as rāhui (Pihama & Lipsham, 2020). Teachers have been seen as “quiet heroes” (Mutch, 2020, p. 7) also serving at the forefront of supporting families and children during the current pandemic. The suddenness of the first national lockdown, imposed in response to the crisis of the rapidly spreading virus, was unprecedented and required immediate adaptation of longstanding teaching practices and pedagogies.

## Government Wellbeing Policies

The role of government in supporting the wellbeing of its citizens is influential, although the nature and extent of this depends on the politics of the day. In 1938, the first Labour government of New Zealand, led by Prime Minister Michael Joseph Savage, responded to the dire situation of widespread unemployment and poverty generated by the Great Depression with the 1938 Social Security Act. This introduced a progressive and comprehensive social welfare system of ‘cradle to the grave’ social security which included a public health system, housing, superannuation, pensions and the promotion of the innovative pedagogies of the international New Education Fellowship (Campbell, 1938; Matthews, 2002; Mutch, 2013; Weijers & Morrison, 2018). Uncharacteristically perhaps, it was the fourth Labour Government of Prime Minister David Lange that from 1984 introduced the neoliberal economic policies that have successively undermined the platform of social welfare which had been associated with Labour governments since the 1930s. Lange’s Government “broke radically from the traditions of the past, starting a process of social and economic reforms [via an] extensive programme of deregulation and privatisation [which] emphasised the role of market forces and markedly reduced both the welfare state and the direct role of the state in the economy” (Blaiklock et al., 2002, p. 1). These and subsequent neoliberal policies, layered upon the legacy of colonisation, have had a longstanding negative effect on the wellbeing of both Māori and children as seen in poverty and suicide statistics (Dale et al., 2011; Pack et al., 2016; Peters, 2013).

## Wellbeing as a Government Policy Aspiration

The 2019 Wellbeing Budget of the sixth Labour government, led by Jacinda Ardern and in the first term in coalition with Winston Peters’ New Zealand First party, signalled a return to progressive policy roots. This built on work that had previously been commenced by the New Zealand Treasury (Weijers & Morrison, 2018). The Wellbeing Budget was intended to address “New Zealand’s long term challenges of mental health, child poverty, children in State care, family violence and homelessness” (Ardern, 2019, pp., para. 1). Ardern, with the support of the then New Zealand First Minister for Children Tracey Martin, was determined to address the significant issues of child poverty as a key wellbeing issue, stating that “New Zealand can and should be the best place in the world to be a child, but we must think about what that means from a child’s perspective” (Ardern & Martin, 2019).

**Fig. 1:**
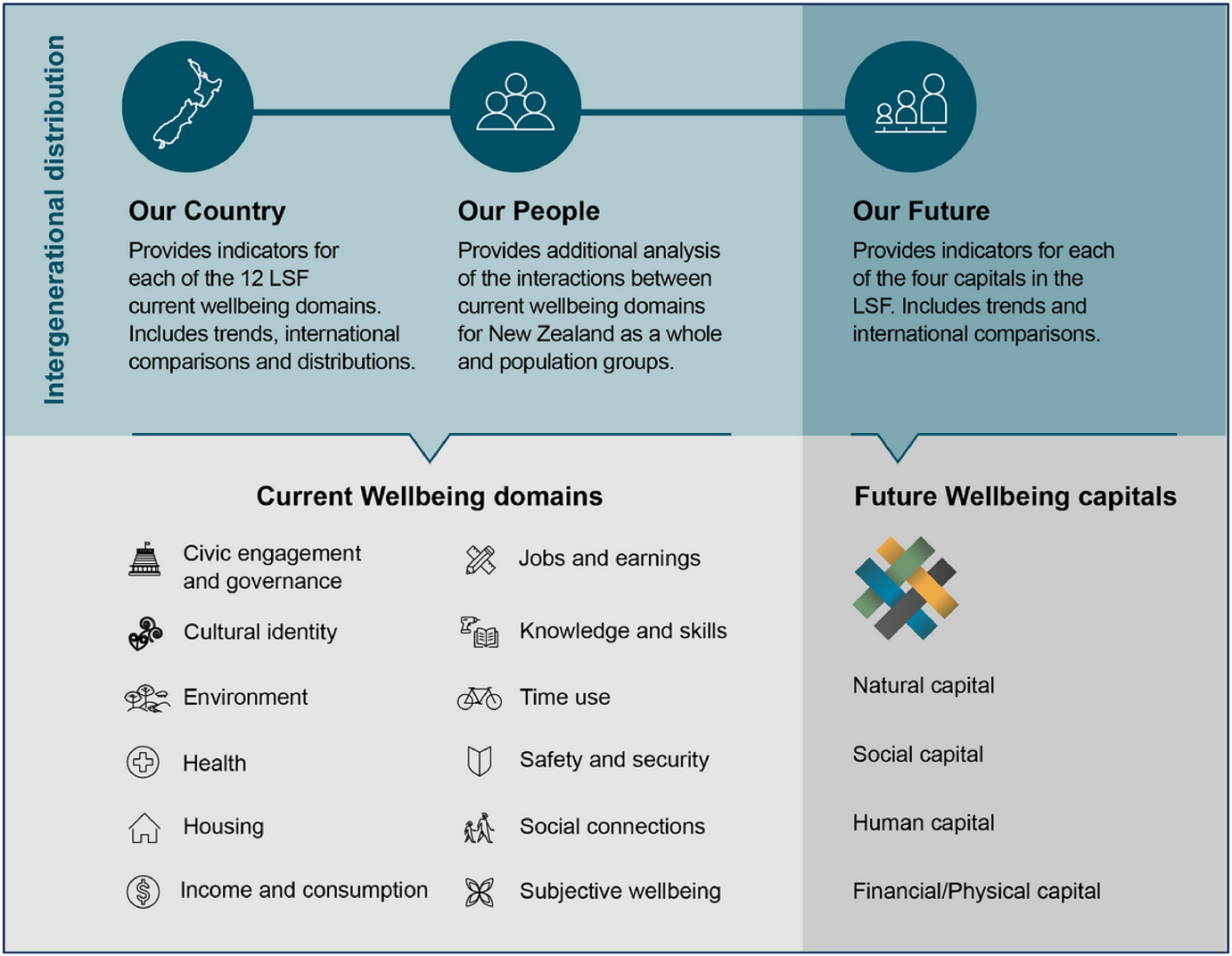
Measuring wellbeing: the Treasury’s Living Standards Framework Dashboard (Te Tai Ōhanga | The Treasury, 2021b)

**Fig. 2:**
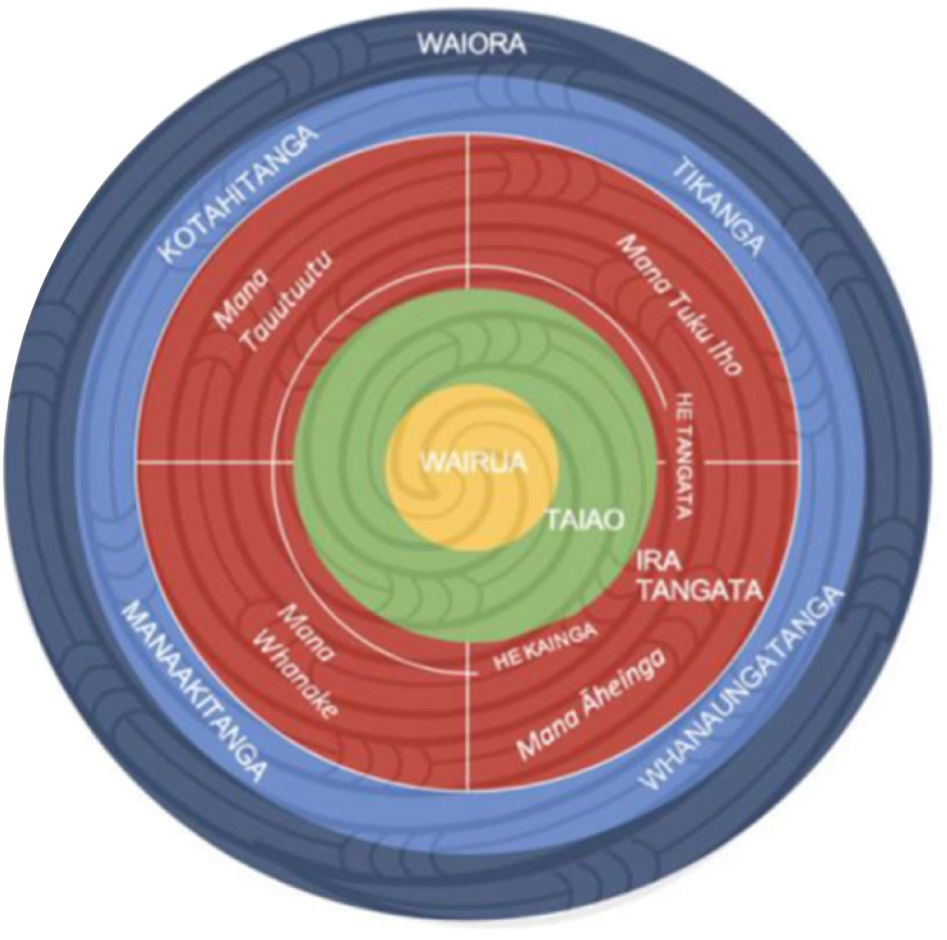
He Ara Waiora model (Te Tai Ōhanga | The Treasury, 2021b)

Wellbeing as a government priority was embedded in Treasury’s ‘Living Standards Framework’ which incorporates a dashboard of purportedly measurable indicators which include civic engagement, environment, cultural identity and ‘subjective wellbeing’ (Te Tai Ōhanga | The Treasury, 2021b) (Fig. 1). The work of Tai Ōhanga | The Treasury has been informed by te ao Māori perspectives such as those outlined in the work of Professor Mason Durie (2006). The Treasury approach is this regard is encapsulated in its He Ara Waiora framework “that helps the Treasury to understand waiora, a Māori perspective on wellbeing” (Te Tai Ōhanga | The Treasury, 2021a, para. 1). It is described as providing “an indigenous and uniquely Aotearoa New Zealand response to these questions by taking a tikanga-based [based in Māori ethics] approach to wellbeing” (Te Tai Ōhanga | The Treasury, 2021b, para. 3) (Fig. 2).

He Ara Waiora identifies the following goals:

**Wairua** (spirit) is at the centre to reflect that it is the foundation or source of wellbeing. Values, beliefs and practices related to wairua are essential to Māori conceptions of waiora.

**Te Taiao** (the natural world – the environment), is paramount and inextricably linked with human wellbeing. Humans have responsibilities and obligations to sustain and maintain the wellbeing of Te Taiao.

**Te Ira Tangata** (the human domain) encapsulates human activities and relationships, including the relationships between generations. The concept of mana (power, authority) is seen as key to wellbeing. (Te Tai Ōhanga | The Treasury, 2021b, para. 7)

In addition, Te Tai Ōhanga | The Treasury lists the following principles of He Ara Waiora:

**Kotahitanga** – working in an aligned, coordinated way

**Tikanga** – making decisions in accordance with the right values and processes, including in partnership with the Treaty partner

**Whanaungatanga** – fostering strong relationships through kinship and/or shared experience that provide a shared sense of wellbeing

**Manaakitanga** – enhancing the mana of others through a process of showing proper care and respect

**Tiakitanga** – guardianship, stewardship (e.g. of the environment, particular taonga or other important processes and systems). (Te Tai Ōhanga | The Treasury, 2021b, para. 8)

Significant in this model is the centrality of wairua, or wairuatanga, the spiritual interconnectedness that pervades all interconnections and inter-relatedness, surrounded by te taiao, the natural world, which is recognised as the key source of all wellbeing.

## Māori and Social Policies

The obligation to prioritise the wellbeing of its citizens is increasingly acknowledged internationally as a key focus for government policies (Weijers & Morrison, 2018) as a counter to the ‘economy first’ focus of neoliberalism. Yet Aotearoa New Zealand has a particular history in that since 1975 successive governments have begun to address longstanding but previously ignored obligations to Māori, the Indigenous people, as stipulated in the 1840 Tiriti o Waitangi (Walker, 2004). Article One confers the responsibility of good governance on the Crown. Article Two of Te Tiriti o Waitangi expresses Crown commitment to tino rangatiratanga, or self-determination by Māori, Article Three assures Māori of their equal citizenship rights, and the fourth verbal article assures Māori that their spiritual belief systems have equal status with those of the two Christian religious denominations whose missionaries were present at the initial treaty signing (Waitangi Tribunal, 2014).

Progressive policies that reflect Tiriti obligations to Māori via incorporating te ao Māori understandings and commitments in social and economic priorities are becoming increasingly prevalent. A ground-breaking education example is the 1996 promulgation of the first bicultural national education curriculum*, Te Whāriki. He whāriki mātauranga mō ngā mokopuna o Aotearoa: Early childhood curriculum* (Ministry of Education, 1996). Further progressive policies have followed in the educational arena, including the recent commitment that New Zealand history is now to be taught at all levels of schooling (Ardern & Hipkins, 2019), alongside a strong focus on addressing historical and contemporary racism, and the strengthening of the teaching of te reo Māori (Ministry of Education, 2019, 2020b, 2020c, 2021).

He Ara Waiora, the model developed by Te Tai Ōhanga | The Treasury (2021b) is an example of how Tiriti obligations towards Māori, as reflected in key Māori values, might be positioned as integral to measuring wellbeing outcomes. As has been recognised, ‘While the principles of He Ara Waiora are derived from mātauranga Māori [Māori knowledge], many of its elements are relevant to the wellbeing of all New Zealanders’ (Cook et al., 2020, p. ii). It has further been argued that it is timely to acknowledge, as with a Māori worldview, that the wellbeing of the whenua (land) or more broadly Papatūānuku, the Earth Mother, is intrinsically related to human wellbeing (Moewaka Barnes & McCreanor, 2019). It can therefore be further argued that affirming and enacting such important central Māori values as have been recognised in He Ara Waiora, and which have also been previously well described elsewhere (Mead, 2003; Royal, 1998) are a pathway for delivering wellbeing, not only for Māori but for other citizens, since what is good for Māori is also good for the nation (Penetito, 2011; Skerrett, 2010) and in fact, also for planetary wellbeing (Joseph et al., 2019).

## The Teaching Profession in Aotearoa and the COVID-19 Pandemic

The COVID-19 pandemic reached Aotearoa early in 2020. Responding to the rapidly developing situation, Prime Minister Jacinda Ardern announced a series of alert levels which culminated in the first ‘level four’ national lockdown with schools and other educational facilities closed from 26 March to 27 April, 2020 (RNZ, 2020, 2021). Extended periods of localised ‘level 3’ restrictions followed, particularly in our largest city Auckland, and a further national ‘level 4’ lockdown took place from 18 August to 1st September 2021 (McGuinness Institute, 2021).

Our education system was seemingly ill-prepared for the sudden transition and concomitant stresses. A 2018 survey, the Varkey Foundation Global Teacher Status Index, which canvassed 1000 members of the public and ‘up to’ 200 teachers in each of 30 countries, found that New Zealand teachers had the biggest workloads in the world, averaging 52.1 h per week (Kirkness, 2018). Interestingly, the survey also revealed that in 2018 New Zealand parents were “less likely to encourage their children to become teachers” than in 2013 (Kirkness, 2018, para. 5). A 2019 New Zealand survey commissioned by the teacher unions of 169 primary and 201 secondary teachers and principals who had left the profession, found that they left mainly due to “a lack of work/life balance and burnout from high workload’ (SchoolNews, 2019, para. 1).

Given this context, it was inevitable that there would be significant impacts for the teaching profession in responding to COVID-19. Whilst the Ministry of Education began providing support, this was not always timely or equitably directed (Hunia et al., 2020; Thornton, 2021). Despite some teachers being expected to provide on-site classes for the children of essential workers during the ‘level 3’ restrictions, teachers were not themselves categorised as ‘essential workers’ (Cherrington, 2021; Ministry of Education, 2020a; Smith, 2021). There was no mention either, of offering priority to teachers in the government’s 2021 COVID-19 vaccination roll-out plan, despite many teachers serving families of essential workers, and thus at greater risk of exposure (Unite against COVID-19, 2021). These policies are indicative of a lack of recognition of the importance of the teaching impression to the wellbeing of our communities, and of the undervaluing of the profession.

## Methodology

In March 2020, in response to growing awareness of the need to understand the implications of the impacts of the pandemic on society, education, and teachers, Associate Professor Louise Phillips called together an international group of scholars (from Australia, New Zealand, Singapore and the USA) to plan a study (L. Phillips et al., 2021a, 2021b). A qualitative survey was collaboratively prepared, using the Qualtrics platform, in order to gather the perspectives of teachers. Ethical approval was obtained from the Human Research Ethics Committee of James Cook University, Australia. The survey contained 22 questions.

We considered that a qualitative, narrative approach within our Qualtrics survey format would best provide rich understandings and examples of ways in which the pandemic was influencing teaching, learning and wellbeing. The initial six questions sought demographic information and the remaining 14 were open-response, as follows:

7. How has COVID-19 impacted your teaching & learning?
8. What is different about your delivery?
9. How has the children’s/students’ learning been affected?
10. How are you addressing diverse learning needs and approaches, cultural relevance and cultural responsiveness in the altered practices to teaching and learning?
11. What are children/students’ questions and concerns and how do you address them?
12. What are the issues you are struggling with and need support with?
13. If you have moved your teaching online what platform/s are you using?
14. If you are teaching online what have you changed in regard to your teaching, to support students?
15. What are the strategies that your students are using to study online?
16. Who and what are your key knowledge sources for teaching remotely?
17. What new partnerships have you formed to deliver teaching and learning?
18. What innovations have you forged or experimented with?
19. Please share a story of a successful teaching & learning encounter. Include a unique url (e.g., dropbox page, Instagram page) with max. of 5 images of artefacts as relevant. Please make sure images have no identifying features (e.g., no people, no local signs)
20. What have you learnt about yourself and your teaching?
21. What helps you get through each day?
22. What do you think your students have learnt broadly about these changes (such as about humanity, about themselves as learners)?

We recruited participants by circulating information about the survey, including the survey link, in various networks to which we had access. In Aotearoa, these included but were not exclusive to the networks of NZEI Te Riu Roa, the main teacher union for early childhood and primary teachers, and He Whānau Manaaki, the regional kindergarten association for the lower half of the North Island. We also invited teachers to share the link to the survey with colleagues, in order to increase the pool of respondents via snowball sampling (Goodman, 2011). The survey was available through an online link from the 4th of May, 2020 and we had received a total of 635 responses before we closed off the survey at the end of 2020.

In Aotearoa, the opening of the survey came just towards the end of the period of mobility restriction, or ‘lockdown’ from the 23rd of March to the 13th of May (RNZ, 2021). During the ‘level 3’ latter period of this lockdown, from the 28th of April to the 13th of May, some early childhood services and schools were opened for the children of ‘essential workers’. This paper draws on the data from the Aotearoa New Zealand respondents (n = 51), which was received in the two-month period from 4th May–3rd of July 2020 (there were no further New Zealand responses after that date). Data were coded using NVivo, an online qualitative software tool, with analysis supported by utilising visual tools from this application, as illustrated later. This is admittedly just one attempt to present data from the survey, and limitations are acknowledged, such as the difficulty in representing a vast amount of qualitative data. Further papers may make a more nuanced exploration of the responses to specific questions as outlined above.

## NZ Data: Demographics

Respondents came from across the entire country, including urban and rural areas of both main islands. The majority, (58.82%, n = 30) were primary school teachers. There were markedly fewer early childhood teachers (11.76%, n = 6) and secondary teachers (13.73%, n = 7) (please see Table 1). The sample included a wide geographical coverage. The ‘other’ category included teachers working in the following roles and sites: learning support, special education, a resource teacher, an Area School (which covers both primary and secondary levels), an intermediate school, the Ministry of Education, and tertiary education. Across all 51 of the respondents there were listed a total of 74 qualifications, which included diplomas (17.57%, n = 13), degrees (40.54%, n = 30), post-graduate qualifications (37.84%, n = 28) and doctoral degrees (4.05%, n = 3). The majority of respondents had been teaching for an extended number of years, half (n = 26) of the respondents for more than 21 years and a quarter (n = 13) for between 16–20 years. None were beginning teachers in their first two years of teaching. (Please see Table 2). In keeping with their reported number of years of teaching experience, almost all of the teachers were aged between 35 and 64. (Please see Table 3).

**Table 1:** Teaching settings

No	Answer	%	Count
1	Early Childhood education settings (e.g., preschool, kindergarten, childcare)	11.76	6
2	Primary School	58.82	30
3	Secondary school/high school	13.73	7
4	Tertiary education	1.96	1
5	Other	13.73	7
	Total	100	51

**Table 2:** Length of experience as a teacher

No	Answer	%	Count
1	Less than 1 year	0.00	0
2	1–2 years	0.00	0
3	3–5 years	7.84	4
4	6–10 years	5.88	3
5	11–15 years	9.80	5
6	16–20 years	25.49	13
7	Over 21 years	50.98	26
	Total	100	51

**Table 3:** Ages of participating teachers

No	Answer	%	Count
1	20–24	0.00	0
2	25–34	7.84	4
3	35–44	25.49	13
4	45–54	31.37	16
5	55–64	31.37	16
6	65–	3.92	2
	Total	100	51

## Qualitative Data—Visual Representations

The team of researchers involved in the wider international study were determined to experiment with various innovative software programmes that might help bring our study to life through the capacity of tools such as Omeka, NVivo, and Voyant, in order to offer new ways of connecting, collaborating and visually sharing our insights (Campbell & Coleman, 2020). Kakali Bhattacharya has highlighted “the depth of inquiry and analysis that can be achieved using software with efficient processes such as clustering, connecting, interrogating data, and visualizing results” (2015, p. 2). In this spirit of inquiry and exploration, this section of the paper has employed some tools from the qualitative data analysis software NVivo to display aspects of the data. It should be noted that this is a tentative exploration of a limited range of NVivo’s possibilities, and also that the static visuals in this section whilst seemingly superficial, could be delved into further, in order to identify relevant data excerpts, as seen in the discussion of Fig. 6.

**Fig. 3:**
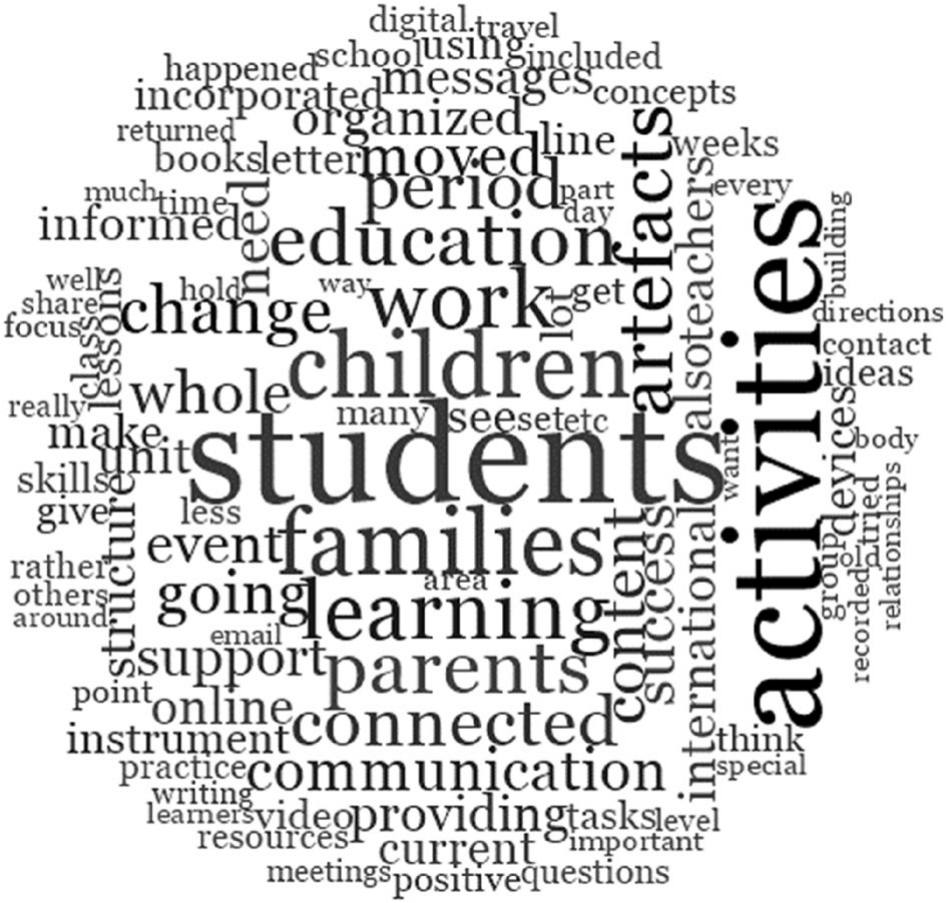
Qualitative data wordcloud

Firstly, the following word-cloud was generated (Fig. 3). This tool juxtaposes commonly utilised words by size, the largest sizes representing the most frequent occurrences. The word-cloud unsurprisingly centred ‘learning’ as the most prominent construct, surrounded in descending order of prominence, by ‘students’, ‘families’, ‘activities’, ‘children,’ and ‘learning’ (please see Fig. 3). Interestingly, in the data from the wider international study, ‘students’ was also the key priority of teachers as depicted in a Voyant ‘TermsBerry’ of the 624 teacher responses to question 12, ‘What are the issues you are struggling with and need support with?’ (L. G. Phillips et al., 2021a, 2021b).

**Fig. 4:**
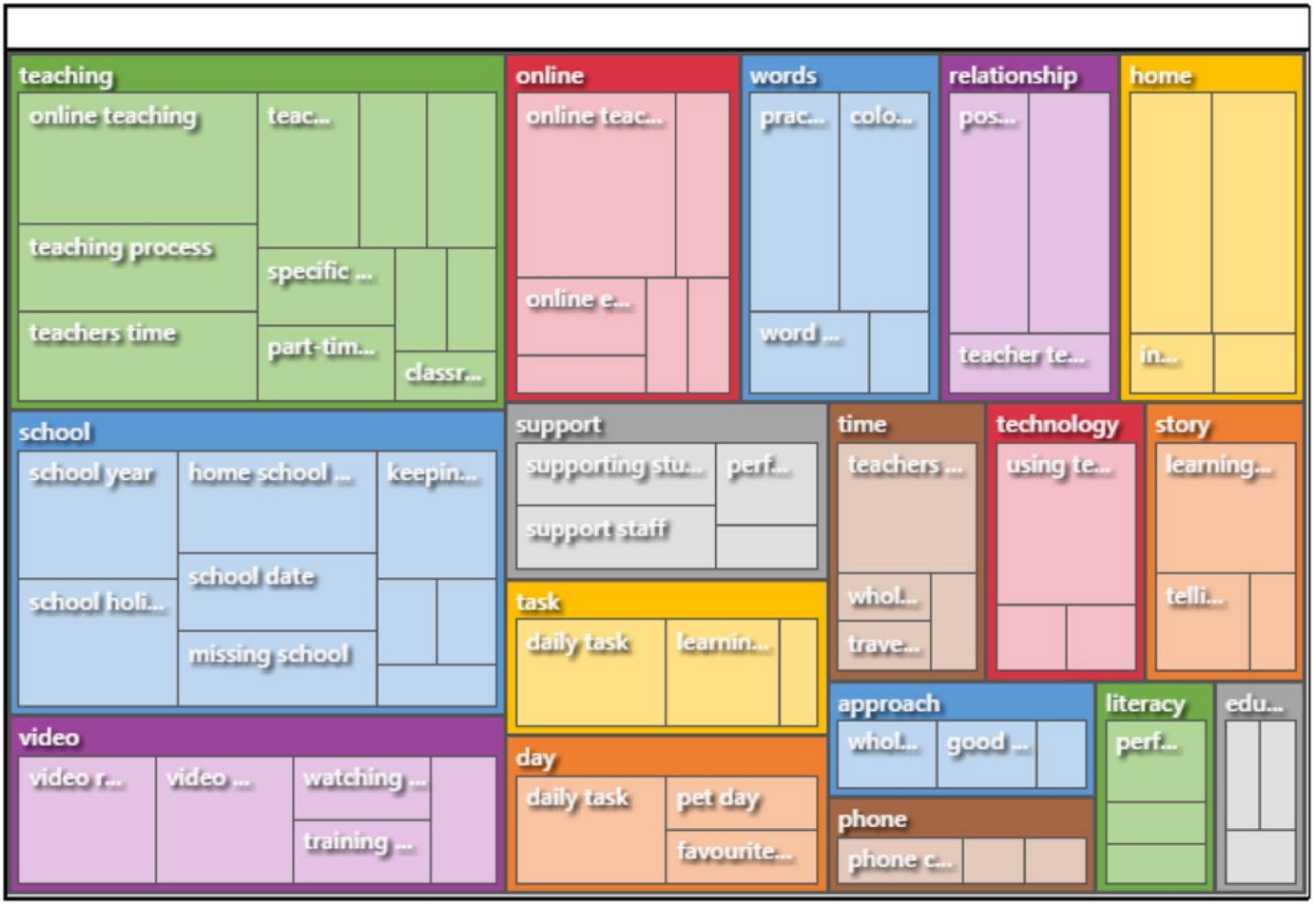
Auto-coded themes, grid format

**Fig. 5:**
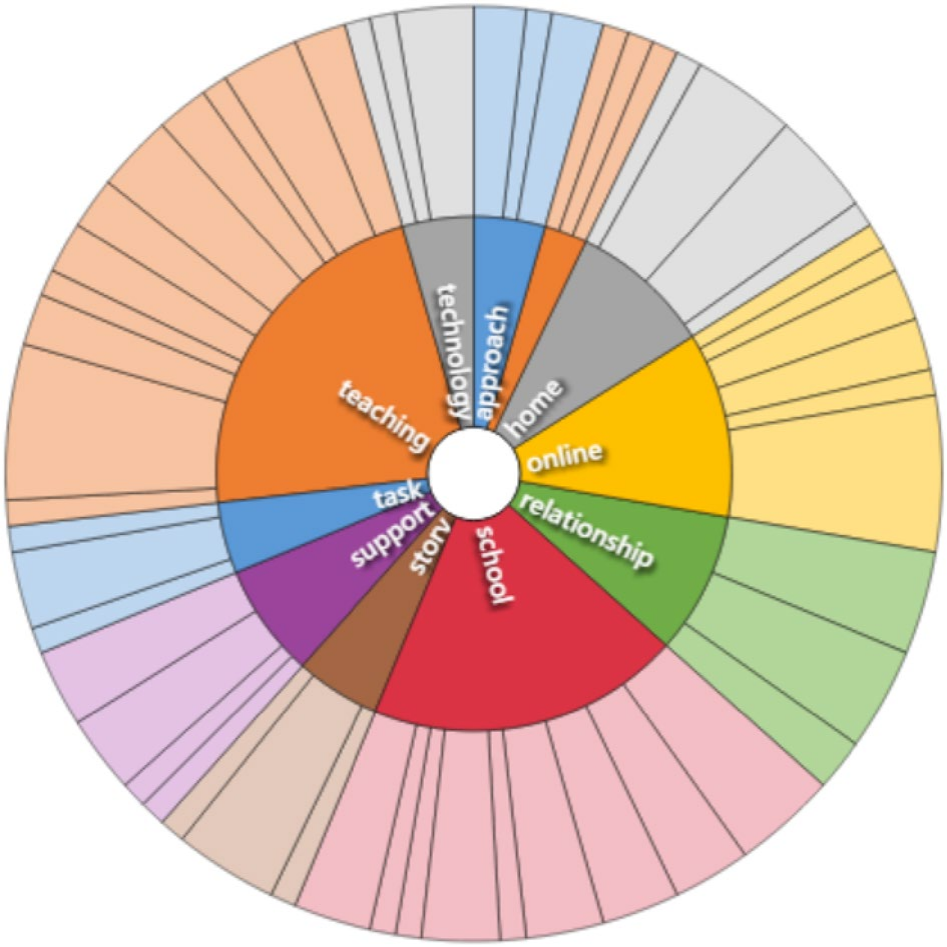
Auto-coded themes, wheel format

In my previous studies NVivo was coded manually, sentence by sentence, or section by section, a slow and methodical and obviously very subjective process. For this paper I explored the use of auto-coding (Chaturvedi & Bansal, 2022). Auto-coding can be a useful tool to explore what concepts and patterns emerge from the data (Kalpokas & Radivojevic, 2022). Figure 4 is an image of the auto-coded theme-set that was generated with automatic colour coding. Within the programme, clicking on the various boxes expands the contents in more detail. For example, opening ‘relationships’ enables further details as to the numbers of data excerpts coded in each sub-category of ‘positive relationships’, ‘teacher-teacher relationships’ and ‘home-school relationships’. Figure 5 shows a further representation of the main auto-coded themes. In both Figs. 4 and 5 the sizes of the respective segments represent the number of data auto-coded to each.

**Fig. 6:**
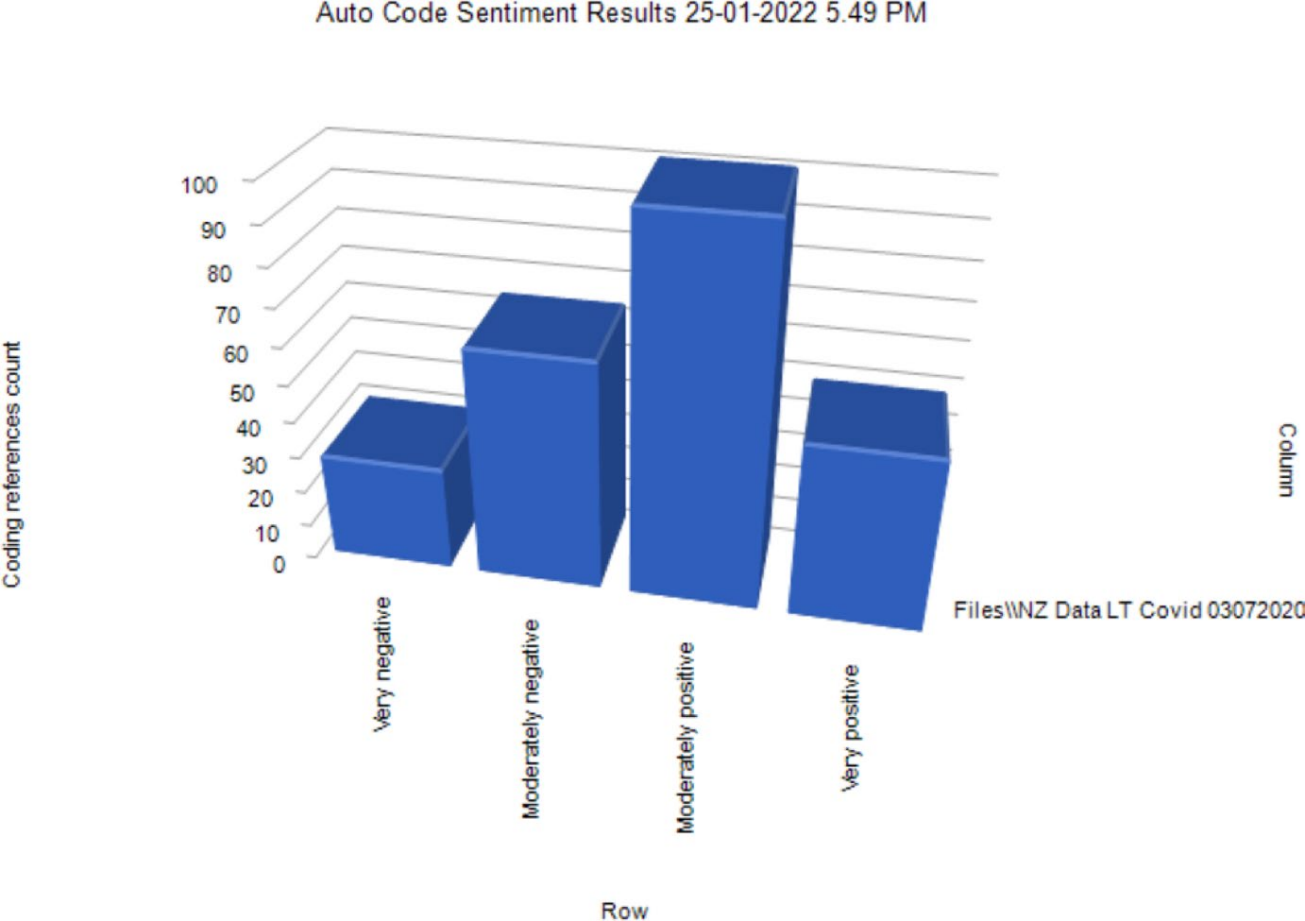
Auto coded ‘sentiment’ results table

Next, an NVivo auto-coding for ‘sentiment’, which indicates a spread of emotions (Chaturvedi & Bansal, 2022), produced the following diagram, Fig. 6, which represents 29 entries coded as very negative; 63 coded as negative; 100 as moderately positive; and 48 as very positive:

This representation offers an interesting visual portrayal of ‘sentiment’ as auto-coded by the NVivo software. Drilling down using the visual model provided examples of data. Some excerpts from the 29 coded as ‘very negative’ included:

It was hard to reach out to families who have no phone number, email, internet or device. This meant that the students that were in this situation (during Level 4) did not get any learning material whatsoever. I have had to adjust to online teaching; video calling my students three times per week, sometimes extra for those that need help or additional support. I found myself always worrying about them as their well-being to me was more important in general. Students have become groggy, tardy and 1pm seemed to be too early for some students for some video calls / online teaching!

Loss of focus, loss of momentum.

Many of our families are struggling financially, some have lost jobs. we have set up a pātaka - donations of food to give to our families. We also really struggle with digital access and are trying to find ways through this. We also have some families whose children have still not returned to school- we are working on this currently.

Included within the 48 examples coded as ‘very positive’ were the following:

We have coped incredibly well to be honest - thanks to an awesome team and a great Learning Support Tool in Seesaw

Reformation of priority areas of learning. Realisation of how indigenous identities, knowledge, pedagogy and wisdom is appreciated. Wellbeing of the learning community is on top shelf. Holistic approach to learning is recommended! Decolonising of current education systems to serve each country's context in relation to its residents.

At senior level, NCEA demands constricted what I could do, but I did my best to provide a variety of resources (including video) so that students were able to engage with content in a variety of ways. With my Year 9 guitar cohort I basically threw away the classroom programme (before lockdown I found out how few of them had a guitar at home!) and found a variety of creative tools online that they were able to create with - virtual instruments, melody and rhythm writing programmes (thinking ahead to the Year 10 programme).

Our students are non-verbal but we have been sharing a lot of resources with parents to support their wellbeing

The visuals derived from NVivo’s auto-coding produced some unanticipated representations. These were useful in that this meant that the focus of analysis was broadened beyond the researcher’s presumptions. For example, NVivo’s coding of ‘sentiment’ as illustrated in Fig. 5 suggests that although overall teachers had a mixed experience of teaching during the first months of the 2020 COVID-19 pandemic, many were resilient enough to focus on positive aspects to the extent that these dominated their reporting.

**Fig. 7:**
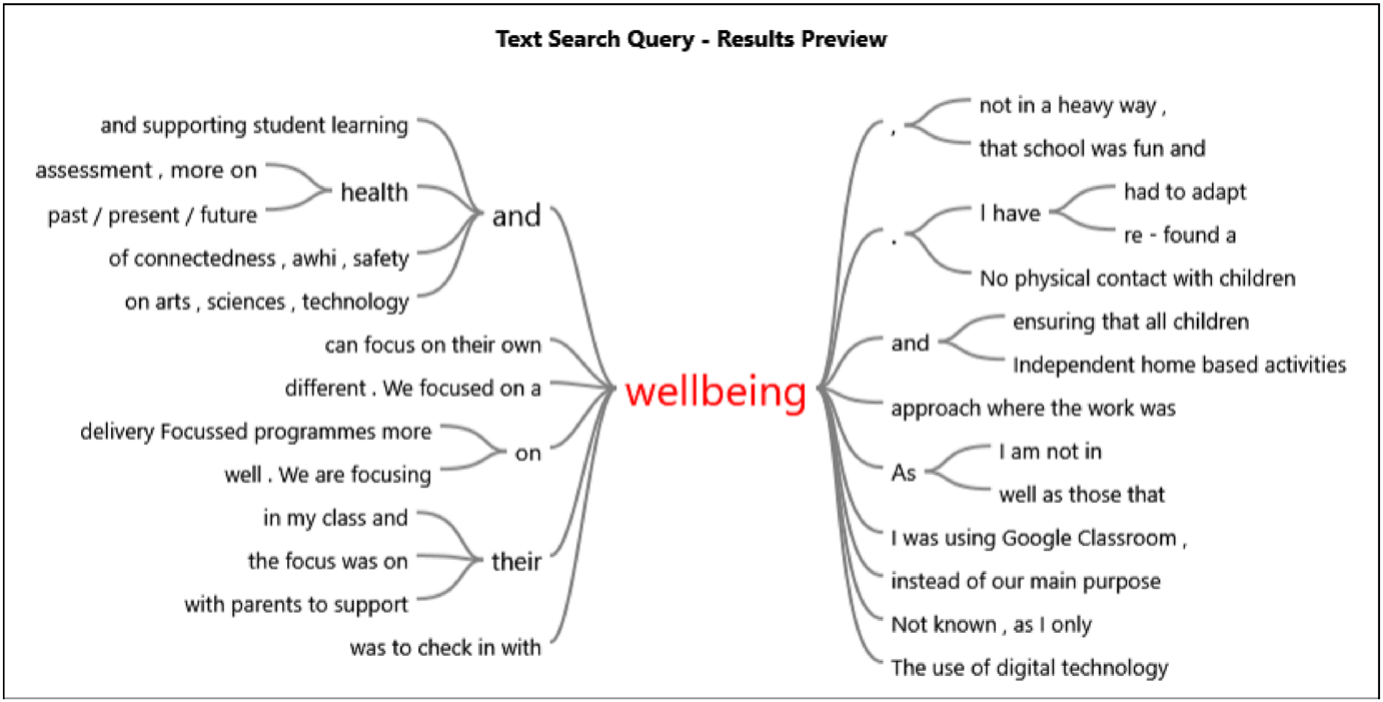
Word frequency relationships query ‘wordtree’ results for ‘wellbeing’

The last visual representation comes from running an NVivo word-tree tool frequency relationships query focussed on the word ‘wellbeing’. (Please see Fig. 7).

The result of this particular NVivo query (Fig. 7) demonstrates some of the ways in which the word ‘wellbeing’ featured in the New Zealand teachers’ data. This leads into the next section in which the voices of the New Zealand teachers are featured.

## Qualitative Data—Voices of Teachers

Building from the generalised pictures presented above, this section provides specific examples of qualitative data from the open-response questions in the survey in order to illustrate ways in which teachers responded to their experiences during this first period of COVID-19 in Aotearoa New Zealand. Many of the teachers noted the challenges of having been required to suddenly pivot to an online teaching environment, with little or no preparation for this situation. In particular, teachers recognised the wellbeing issues affecting the children, and working closely with their families as key to engaging with them in this online context. Teachers also identified inequities with regard to barriers to internet and device access for many children and families:

Covid made me stop and think about how I was going to teach, the impacts this would have on families, the skills and knowledge that families had, access to computers etc. The normal way of teaching stopped.

With the lockdown we were expected to teach online. Because of the low level of ICT literacy it was quite different. We focused on a wellbeing approach where the work was set and it was optional - This was to take away stress from parents and caregivers. I also had daily video chat opportunities for children to catch up and to check in. I read from a novel each day to create a stimulus to keep children coming back each day.

Despite these access issues, teachers worked at employing various strategies for becoming more connected with children’s families during the period of lockdown online teaching, demonstrating sensitivity to their presence in diverse families’ homes as seen in the following examples:

Working from home meant planning learning opportunities that were inclusive for all my students and easy for parents to deliver/assist. I included more digital learning in the programme. Greater communication and relationships built with whānau [families].

This has been very important to us as we have the view that through the digital technology whether we are using Google Meet, Zoom or other ways we are actually visitors into the children’s homes. Families have responded really well and often it is the parents who just want to see the teacher or teacher aide as much as the children do. Respectful and warm relationships are key.

The activities I set allowed for and encouraged families to adapt and share from their backgrounds.

I translated documents into Chinese. With permission I shared a video with the class about colour words in Samoan submitted by a student with a Samoan parent. I included links to Te Reo Māori [Māori language] videos and worksheets in my initial package to parents.

Routine use of karakia and whakatauki [Māori rituals of spiritual acknowledgement; Māori wisdom sayings]. One-on-one sessions with my Autistic boy - this has also meant more family contact via online class where the parent sits in, and also more phone calls. A big part of the online class was to check in with wellbeing, not in a heavy way, but just using humour, etc. Making sure to talk to any siblings join in with us, acknowledging any family members, pets they may show on the screen etc - trying to enter their world. Getting students to share funny activities via photos and captions. Taking time to find out more of their interests and encouraging them to use the time to explore them.

Our students are non-verbal, but we have been sharing a lot of resources with parents to support their wellbeing.

Some teachers felt that their online pedagogies had improved their relationships with families, as well as benefitting the students.

I have developed more of a positive relationship with most of my families and now feel that I can contact them for any issue that I need as they are with me. The home school relationship has strengthened and become more open within my class.

Mokopuna [children] still learnt and developed. Whānau [families] know them best and they had wonderful experiences at home and made many memories.

Most of the students I work with benefited a lot from their parents’ 1-1 input.

I have found that many parents have identified that they need to support their child more in areas from what they are seeing and hearing. There are now no surprises in how their child is progressing.

It was evident that many teachers recognised the need to prioritise their students’ wellbeing at this stressful time of rapid change and uncertainties. One teacher explained that “For us the focus was on their wellbeing, that school was fun, and creativity has reigned”. Another teacher identified a shift in priority from “Less emphasis upon assessment, [to] more on health and wellbeing” and a further response noted barriers to students’ access to learning, along with concerns about their wellbeing becoming a key focus:

It was hard to reach out to families who have no phone number, email, internet or device. This meant that the students that were in this situation (during Level 4) did not get any learning material whatsoever. I have had to adjust to online teaching; video calling my students three times per week, sometimes extra for those that need help or additional support. I found myself always worrying about them as their well-being to me was more important in general. Students have become groggy, tardy and 1pm seemed to be too early for some students for some video calls / online teaching!

Teachers articulated underpinning values of kindness, empathy, connection and compassion as key to supporting their students through this stressful initial period of the pandemic:

My delivery was also more relaxed and friendly due to trying to deliver in a way that would not cause students to become anxious or put them under undue pressure because of the situation.

I sent every child a letter and made them a kindness mouse where they could tie a knot in the mouses tail every time they did something kind. Parents were pleased more for these types of communication and interaction rather than pushing the learning and academic side of things as the children were learning in a different way and about how to be kind.

People need connection and it is important to find ways to maintain that connection.

With compassion, patience and understanding.

With care, concern, reflection, passion and compassion.

Calmly with confidence. Children are suffering from too many negative messages. They need hope.

For those teachers who held roles as education leaders, there was an additional focus on coordinating supports for teachers, families and students, again with a wellbeing focus:

As I am not in the classroom it has been from the aspect of leading a staff through the teaching and learning of their classes and planning for the best possible outcomes for learners and their families through this time. This has included support staff and other adults who work close to the children including the Social Worker in schools, the public health nurse, caretaker, Resource Teacher of Māori and part-time teachers. It has been important to find ways for people to stay connected, feel well supported in new ways of delivery, feel like their contributions matter, receive appropriate PLD [professional learning and development] and feel that they can focus on their own wellbeing as well as those that they teach. As airlines would say - it has been important to put your own oxygen mask first.

Exhausted - being a teaching principal with all the extra workload in relation to extra paperwork etc.. and being the main person contacting whānau and keeping school community informed and supporting student learning and wellbeing.

In addition to the steep learning curve involved in adapting to online teaching, some teachers reported challenges to their own wellbeing, such as tiredness, anxiety about their own health issues, eye-strain, and concerns for their students who were unable to access online-learning:

Not being able to connect with all my students, and the lack of the natural relational aspect of face-to-face learning. The difficulty of being able to pitch the learning at a level that is suitable for the age of my students, when the need for differentiation is so high in the classroom. Developing eye strain and fatigue from being online all day. Other concerns are around accessing learning due to lack of suitable devices, this has been mostly rectified by the school sending out devices. Also, the Ministry provided some at home learning packs, but many of my students did not qualify.

After the initial April 2020 national lockdown, the nation was now under ‘level three’ restrictions, which required people to remain at home unless their work role prohibited this. Some teachers were required to return to their schools and centres to support the children of families of essential workers. A kindergarten teacher reported that “Back teaching in kindergarten Alert level 3. Already had systems in place to connect with whānau and mokopuna. Our curriculum became one of connectedness, awhi [support], safety and wellbeing”. The focus on wellbeing persisted beyond the initial period of online teaching during lockdown:

We are focusing on wellbeing and ensuring that all children feel safe and welcome at school before we move our focus back onto reading, writing and mathematics.

It was completely different during the lockdown. Since we have been back at school we have had a wholistic approach to education where we have focused on arts, sciences, technology and wellbeing instead of our main purpose being reading, writing and mathematics. I think this has been a good approach because of the uncertainty in the children's lives and the massive change that happened over night.

Mokopuna [children/grandchildren] are so resilient; they were so excited to be back with kaiako [teachers] and friends. Some know a bit about Covid, so we listen to what they have to share and kōrero in ways that are relevant and meaningful for them.

I think they have come back a lot more relaxed and parents have definitely changed their views on their own child and their education.

These young children have come back from lockdown showing the most amazing empathy towards each other, hugs, affection, helping to soothe and comfort each other.

These examples demonstrate the resilience of teachers, children and families in reacting to, and rebounding from the rapid and intense changes during the initial period of COVID-19 lockdowns. They reflect teachers’ concern for the wellbeing of their students and students’ families, as well as their strategies for seeking support from families in reaching their children. Dispositions of wellbeing were apparent, in their discussion of the increased emphasis on empathy, care, and compassion. Represented here at the individual and community level, the teachers’ responses have implications for future government education and wellbeing policy.

## Implications of Teachers’ Experiences for Future Government Policy

As demonstrated by the teachers in our study, the pandemic required an immediate national (and international) pivot to new ways of thinking, being, doing, and relating. As seen in Fig. 1, the teachers' keywords focused on students, children, families, learning, parents, connection and communication. Less evident were the educational priorities of curriculum, assessment, and measurement of outcomes. Many of the teachers in our survey re-centred their focus on the emotional wellbeing of their students. This shift could be theorised as explaining the perhaps unexpectedly high levels of ‘positive sentiment’ as shown above in Fig. 6. Colleagues who analysed data from higher education participants in the wider survey similarly found that those respondents “perhaps unexpectedly moved purposefully towards understanding, meaning-making, agency, creativity, and hope, with a central emphasis on relationships and a pedagogy of care and kindness” (Cain et al., 2022, p. 15). They concluded that: “Learning through adversity has seen our participants come out the other side as more skilled, empathetic, and creative teachers. Should they be forced to teach online in the future, they will be ready to meet that challenge” (Cain et al., 2022, p. 16).

For many people in Aotearoa, the lockdown experience precipitated the re-setting of priorities, as indicated in a mixed methods Aotearoa study conducted towards the end of the first 2020 lockdown (Officer et al., 2022). Respondents identified aspects of the ways their lives had changed during the lockdown experience that they now wished to retain. These included “living a simpler life, appreciating local community connections, considering the environmental impacts of modern life, finding alternatives to ‘going out’ and commercialisation, and being positive and grateful for what they have” (Officer et al., 2022, p. 10). Authors of a large-scale survey of 3116 Māori participants conducted in 2020 summarised similar aspirations from their respondents:

For many, the pandemic lockdown provided an opportunity for slowing down, thinking and living differently – it was a time of consuming less, and spending quality time with whānau, and reflecting on what really matters. Many indicated a desire to retain this experience post COVID and not return to the way they were before (Houkamau et al., 2021, p. 31)

The revaluation of priorities by Māori in that study included calls for reforms at the level of government economic policies: “Many of our participants called for a country which adopts a different economic mind-set, focusing on producing what we need to live, rather than focusing on consumerism, short term profits and economic growth” (Houkamau et al., 2021, p. 31).

The onset and prolonged duration of the COVID-19 pandemic has certainly provided an extreme test of any government’s capacity to protect the wellbeing of its citizens. In Aotearoa, both the health and education sectors had been consistently under-funded during the period of National-led government from 2008–2017 (Cumming, 2017; Wylie, 2017). Despite the best wellbeing-focussed intentions of the Labour government led by Jacinda Ardern, the pandemic exacerbated pre-existing strains and inequities (New Zealand Infrastructure Commission, 2021). It is now increasingly recognised that we will never return to the pre COVID-19 ‘normal’, and it is imperative that future policies additionally recognise the wider impacts on human wellbeing of the climate crisis, pollution and biodiversity collapse (Cooper, 2022). As such, there is an imperative to address the crisis of social inequities and environmental catastrophe, and in doing so take the opportunity to rethink economic, health, education, and other key policy areas.

The government’s stated commitment to wellbeing, to be measured by Treasury indicators, informed by a te ao Māori world view (Te Tai Ōhanga | The Treasury, 2021a) as in the discussion above of Treasury’s Living Standards Framework Dashboard (Te Tai Ōhanga | The Treasury, 2021b), whilst clearly innovative, needs also to be responsive to challenges such as those posed in responding to the pandemic. As per the obligations of Te Tiriti o Waitangi (Walker, 2004), iwi Māori (Māori people, tribes) should be at the heart of such decision-making, centring Māori wellbeing as key to policy-making (Pihama & Lipsham, 2020). The early childhood and primary teachers’ union NZEI Te Riu Roa has articulated the following challenge to government:

We encourage the Government to take a Māori-first approach in taking up, engaging with, and implementing any of the recommendations below – prioritising Māori thinking, learning and practice. Not only because ‘what is good for Māori is good for everyone’, but because a Māori-first approach is the only way we can honour Te Tiriti while creating an education system that is uniquely ours. (NZEI Te Riu Roa, 2020, p. 3).

## Concluding Thoughts

We hope that insights from this and other articles published from our study (see for example, L. Phillips et al., 2021a, 2021b; L. G. Phillips et al., 2021a, 2021b) will be useful in informing future education policy development and in providing recognition of the dynamic, resilient, dedicated and hard-working education workforce that has supported our nation throughout this pandemic. We suggest that it is time for teachers to be given the recognition, status and incomes that reflect their contribution to the wellbeing and greater good of society. We draw attention to the wellbeing values outlined in the te ao Māori framework He Ara Waiora (Te Tai Ōhanga | The Treasury, 2021b) and to the wellbeing dispositions articulated by the teachers in this paper. The prioritisation of supporting the application of such wellbeing approaches from policy rhetoric into practice is one pathway forward through the portal that this pandemic has created.

## Data Availability

Further information about this study is available here: https://omeka.cloud.unimelb.edu.au/teaching-and-learning-in-a-pandemic/.
